# Cerebrospinal fluid dissemination of anaplastic intraventricular meningioma: report of a case presenting with progressive brainstem dysfunction and multiple cranial nerve palsies

**DOI:** 10.1186/s12883-016-0611-6

**Published:** 2016-05-31

**Authors:** Motoki Fujimaki, Masashi Takanashi, Manami Kobayashi, Kei-ichiro Wada, Yutaka Machida, Akihide Kondo, Nobutaka Hattori, Hideto Miwa

**Affiliations:** Department of Neurology, Juntendo University Nerima Hospital, 3-1-10 Takanodai, Nerimaku, Tokyo, 177-8521 Japan; Department of Neurology, Juntendo University School of Medicine, 2-1-1 Hongo, Bunkyouku, Tokyo, 113-8431 Japan; Department of Neurosurgery, Juntendo University School of Medicine, 2-1-1 Hongo, Bunkyouku, Tokyo, 113-8431 Japan; Postal Address: 2-1-1 Hongo, Bunkyouku, Tokyo, 113-8431 Japan

**Keywords:** Brainstem dysfunction, Multiple cranial nerve palsies, Cerebrospinal fluid dissemination, Anaplastic meningioma

## Abstract

**Background:**

It is extremely rare to see cerebrospinal fluid dissemination of intraventricular meningioma, particularly with the development of acute, progressive brainstem/cerebellar dysfunction with an absence of mass formation in the corresponding anatomical sites.

**Case presentation:**

An 81-year-old man was admitted because of double vision, right facial nerve palsy and truncal ataxia. Brain magnetic resonance imaging showed normal findings except for a tumor mass in the left lateral ventricle, which had been noted over 6 months previously. The patient developed hiccups, hyperventilation, and drowsiness, which worsened progressively, and did not respond to corticosteroid or intraventricular immunoglobulin therapy. Cerebrospinal fluid study revealed a mild elevation of protein, and cytology was negative. The patient died and an autopsy was performed. Postmortem investigation disclosed a malignant transformation of benign fibroid meningioma with cerebrospinal fluid dissemination of the malignant cells, diversely involving the surface of brainstem, cerebellum, and spinal cords, secondarily resulting in extensive ischemia in the brain parenchyma by vessel occlusion.

**Conclusion:**

If a patient with an intraventricular tumor develops acute, progressive neurological symptoms, the possibility that it is be caused by cerebrospinal fluid dissemination of tumor cells, after malignant transformation, should be considered.

## Background

Intraventricular meningioma is a rare neoplasm, representing only 0.5–3 % of all intracranial meningiomas [1]. Moreover, it is extremely rare to see a metastasis of meningioma cells (malignant meningioma), via cerebrospinal fluid (CSF) dissemination, involved in the diverse central nervous system (CNS) structures, such as cerebellum, multiple cranial nerves, spinal nerve roots and the cauda equina [2–4]. Recently, we encountered a patient who developed acute progressive decline of brainstem function caused by the CSF dissemination of intraventricular malignant meningioma. Here, we present the clinical and pathological data of the patient.

## Case presentation

**Fig. 1:**
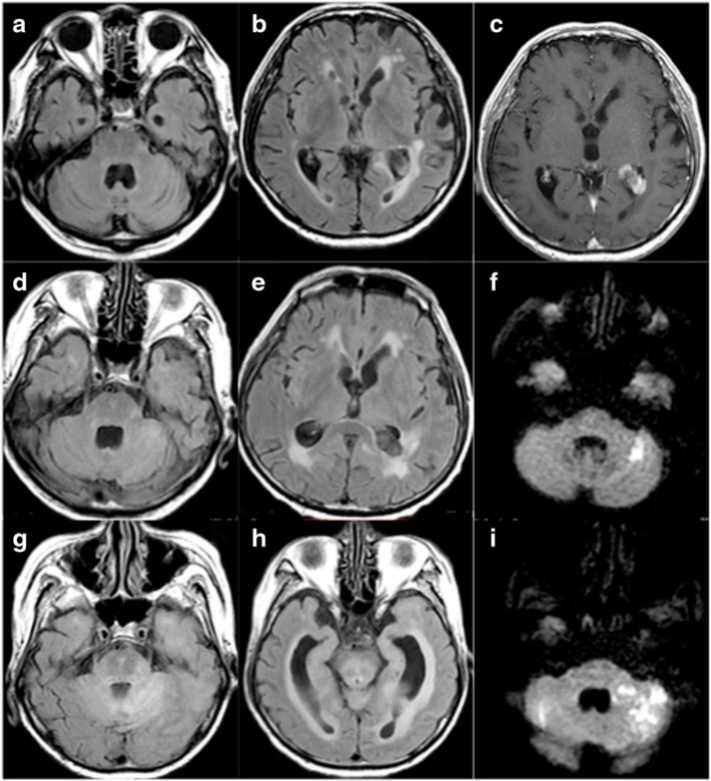
Brain MRI of the present patient at admission (**a –c**), and 17 days (**d–f**) and 40 days (**g–i**) after admission. Fluid attenuated inversion recovery (FLAIR) images at admission showed no gross abnormality, including in the brainstem and cranial nerves (**a**, **b**), although a mass-enhancing lesion was observed in the trigone of the left lateral ventricle on T1-weighted imaging (**c**). 17 days after admission, FLAIR images revealed slight hyperintensity in the exit for the trigeminal nerve and the left cerebellar hemisphere (**d**, **e**), and diffusion-weighted imaging (DWI) showed hyperintensity in the left cerebellar hemisphere (**f**). At the final MRI at 40 days after admission, diffuse high-intensity lesions and hydrocephalus were observed in the brainstem and cerebellum on FLAIR (**g**, **h**) and DWI revealed multiple hyperintense lesions in the bilateral cerebellar hemispheres (**i**)

An 81-year-old man was admitted to our hospital following the appearance of diplopia and facial nerve palsy on the right side. He had noted a mild unsteadiness of gait for 3 months, which he assumed was due to advancing age. There were no extraordinary findings in his medical and familial history. The patient was in good physical condition and without lymphadenopathy. On neurological examination, he had double vision, despite no obvious limitation of extraocular muscles, peripheral facial nerve palsy on the right side, slurred speech, and truncal ataxia. He had no weakness or ataxia of extremities, and his sensation was intact. He had a difficulty in micturition. He had no headache, papilloedema, and consciousness disturbance indicating hydrocephalus. Brain magnetic resonance imaging (MRI) showed expansion of the fourth ventricle and a mass lesion in the trigone of the left lateral ventricle, which was enhanced but with some distortion (Fig. 1a–c). This mass had been noted by chance over 6 months prior to admission when he had medical check-up of the brain and had not changed in size. Laboratory studies revealed that the patients’ blood cell counts and chemistry were almost normal. Soluble interleukin 2-receptor (sIL2-R), angiotensin-converting enzyme (ACE), antinuclear antibody, anti-neutrophil cytoplasmic antibody (ANCA), and tumor markers were all within normal limits. In addition, no anti-ganglioside antibodies or anti-aquaporin-4 (AQP-4) antibodies were present. CSF examination revealed an elevated protein concentration (125.5 mg/dl) and cell count (white blood cell 20 cells/μl; monocyte count, 16 cells/μl) with normal pressure (80 mmH_2_O), but cytology was negative. Because the patient initially developed peripheral facial nerve palsy and a mild unsteadiness of gait, and neuroimaging and laboratory findings were nonspecific or unremarkable, we suspected that he might have an immune-mediated disease such as brainstem encephalitis. Thus, we initiated immunological treatments; these included intravenous methylprednisolone (1000 mg/day, 3 days) and intravenous immunoglobulins (IVIg, 0.4 g/kg, 5 days). Despite these treatments, the brainstem symptoms progressively worsened; 1 week after admission the patient developed persistent hiccups and hyperventilation, followed by lethargy. Fluid attenuated inversion recovery (FLAIR) at 17 days after admission revealed a slightly hyperintense lesion in the exit of the left trigeminal nerve and the left cerebellar hemisphere (Fig. 1d, e). Diffusion-weighted image (DWI) of brain MRI revealed a hyperintense lesion in the left cerebellar hemisphere (Fig. 1f). MRI of the cervical and lumbar spinal cord did not reveal any abnormalities (not shown). Although oral administration of predonisolone (60 mg/day) was continued, the patient’s level of consciousness progressively deteriorated with ataxic respiration and enhanced startle reflex. Simultaneously, his deep tendon reflexes were lost. CSF examination was performed repeatedly, showing mild pleocytosis (white blood cell count 52 cells/μl; monocyte count 51 cells/μl), but the cytology results were negative. FLAIR MRI of the brain, at 40 days after admission, revealed slightly diffuse hyperintense lesions in the left cerebellum and pons with an expansion of the inferior horn of the lateral ventricles (Fig. 1g, h). However, no obvious mass lesions in the cerebral parenchyma or enlargement of the tumor mass in the trigone of the left ventricle was demonstrated. DWI showed multiple infarctions in the left cerebellar hemisphere, pons, occipital lobe, and bilateral corona radiata (Fig. 1i). Fifty-three days after admission, the patient died of respiratory failure, and an autopsy was performed.

**Fig. 2:**
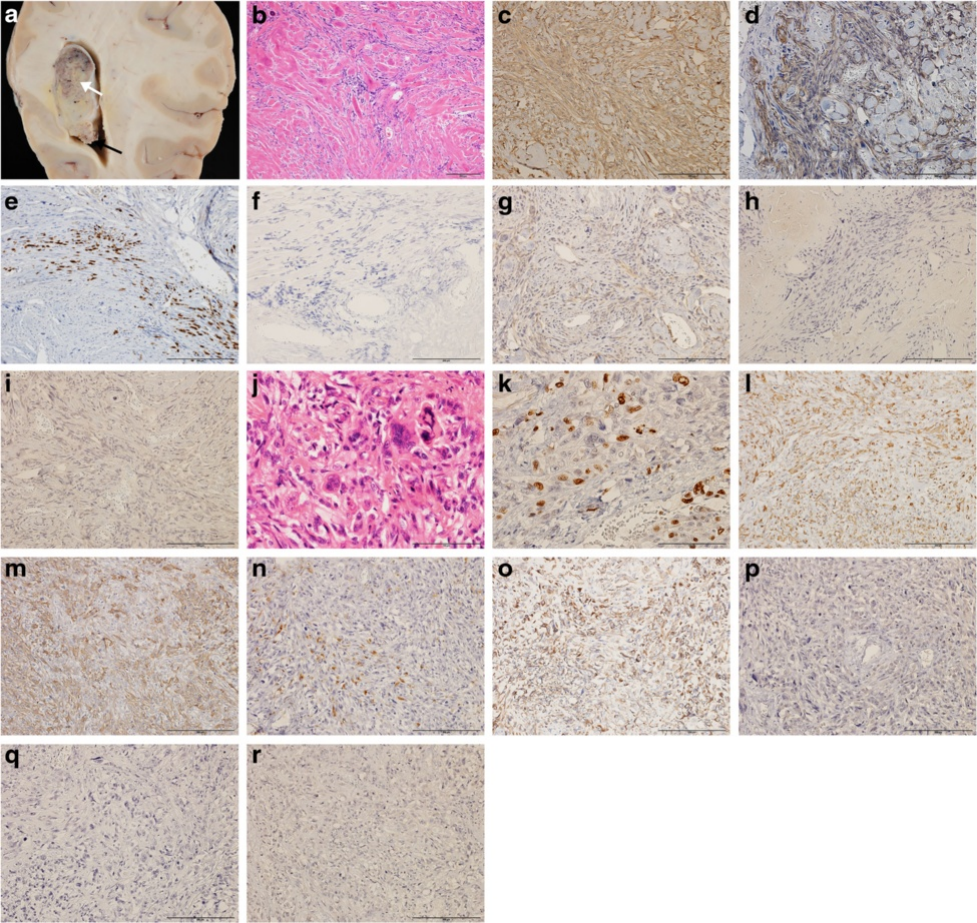
**a**: Macroscopic photograph of the intraventricular tumor. The main part of the tumor was encapsulated and solid (*white arrow*). The bottom part of the tumor became prominent and soft (*black arrow*). **b-i**: Histopathology of the main part shown with a white arrow in Fig. 2a. This region consisted of spindle-shaped fibrous cells, including a large amount of collagen deposits (**b**, hematoxylin & eosin (HE) stain). Using immunochemistry, the cells positively stained for vimentin (**c**), epithelial membrane antigen (EMA) (**d**) and S-100 protein (**e**), but were negative for cytokeratin (**f**), Schwann 2/E (**g**), GFAP (**h**), Olig2 (**i**) and lymphocytic markers (data not shown). These cellular profiles confirmed the histological diagnosis of the tumor as fibrous meningioma (WHO grade I). **j-r**: Histopathology of the part shown with a black arrow in Fig. 2a. This region showed increased cellularity, and cells were large, atypically shaped with multiple nuclei and prominent nucleoli (**j**). Mitotic cells were confirmed by examining more than 20 percent high-power fields on HE (**j**) and Ki67 stain (**k**). The cells with a malignant appearance were also positive for vimentin (**l**), EMA (**m**), S-100 protein (**n**) and cytokeratin (**o**), but negative for Schwann 2/E (**p**), GFAP (**q**) and Olig2 (**r**); This part of the tumor was diagnosed as anaplastic meningioma (WHO grade III)

At post-mortem the major organs showed no pathological changes. In the brain, the tumor in the lateral ventricle was solid and well-demarcated, but the surface of the tumor partly collapsed (Fig. 2a). Histopathological analysis of the intraventricular tumor revealed aggregations of spindle cells including rich collagen deposition and psammoma bodies in the central lesion (Fig. 2b). Additionally, dense cells with a malignant appearance (high nuclear/cytoplasmic ratio, prominent nucleoli, and multinuclear) and increased frequent mitoses were observed at the marginal part of the tumor. The mitotic index was more than 20 mitoses per ten high-power fields on hematoxylin & eosin (HE) and Ki-67 immunostaining (Fig. 2j, k). The cells in both benign and malignant parts were positive for vimentin and epithelial membrane antigen (EMA), partially positive for S-100, but negative for cytokeratin, Schwann/2E, glial fibrillary acidic protein (GFAP) and Olig2, suggesting that the tumor cells originated from a meningioma (Fig. 2c-i, l-r). Thus, the pathological diagnosis of the intraventricular tumor was a mix of fibrous meningioma (WHO grade I) and anaplastic meningioma (WHO grade III).

**Fig. 3:**
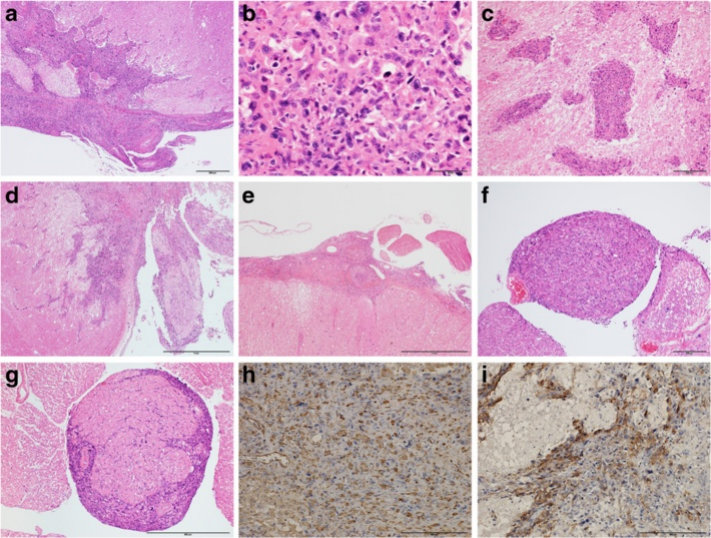
**a-i**: Histopathology of dissemination and infiltration of tumor cells. Copious cells invaded the surface, parenchyma and vessels of the brainstem (**a**, **b**; medulla oblongata, **c**; pontine base, **d**; pons and trigeminal nerve), spinal cord (**e**; dorsal part of T2) and nerve root (**f**; dorsal root of L2, **g**; cauda equina). The cells invaded directly from the surface and damaged the tissue structures (**a**, **d**, **f**). Moreover, vascular infiltration of the cells caused ischemic necrosis around the vessels (**c**). The cells, which invaded the brainstem, pontine, spinal cord and nerve root, also showed the anaplastic meningioma with a highly increased mitotic ratio (**b**) and were positive for vimentin (**h**) and EMA (**i**)

The malignant cells disseminated and invaded the parenchyma and small vessels of the brain – preferentially invading the surface regions of the midbrain, pons and medulla oblongata. Invasion of small vessels resulted in ischemic damage around them. These ischemic changes, including the brain malacia, were found in the cerebellar white matter, pons and lower part of cerebral parenchyma (Fig. 3a, c). Cranial nerves, such as the facial nerve, trigeminal nerve, and oculomotor nerve, were packed with tumor cells (Fig. 3d). Furthermore, tumor cells were diffusely found adhered to the thoracic to lumbar spinal cord and multiple nerve roots, as well as the cauda equina, which were similarly filled with tumor cells (Fig. 3e-g). Because all the observed tumor cells shared the same immunohistochemical characteristics (Fig. 3h, i), it is possible that the copious malignant cells originated from the anaplastic meningioma in the left ventricle of the present patient and disseminated extensively through the CSF, resulting in progressive dysfunction of brainstem and cerebellum.

**Fig. 4:**
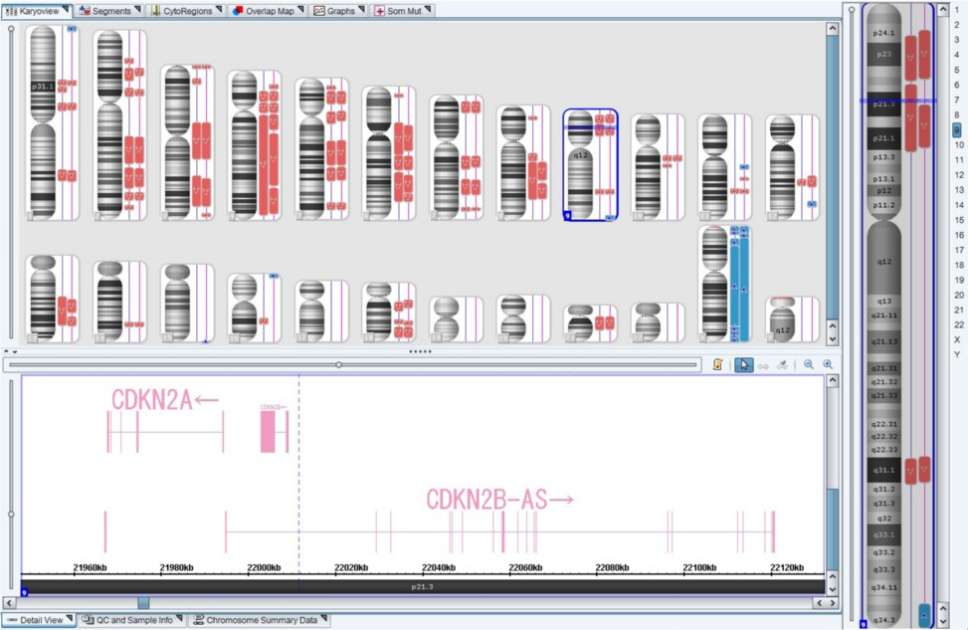
The chromosome analysis for the two tumor parts. The deletion of chromosome 9p21 was detected in the malignant region, the left side sample

We also performed molecular analysis for two regions of the tumor, the fibrous cell part and the malignant cell part. Our chromosome analysis revealed more than 500 differences of gene expressions between two regions. Interestingly, only in the tumor from the malignant region, the deletion of chromosome 9p21 was detected where the genes CDKN2A and CDKN2B were located (Fig. 4). It has been well known that the homozygous deletions of CDKN2A/B are found in most anaplastic meninigomas and these genes have been studied for abnormalities in advanced meningioma [5]. Then, we considered that the malignant region was an anaplastic meningioma transformed from the benign cells, also in molecular aspect. Tumor samples were collected from the autopsy of this patient. Written informed parental consents were obtained prior to sample collection. The study was approved by the institutional review boards of Juntendo University. Genome DNA was extracted from Paraffin tissue blocks using GeneRead DNA FFPE Kit (Cat. No.180134,QIAGEN, Hilden, Germany). The DNA was amplified by REPLI-g FFPE Kit (Cat. No.150243), and was investigated for genomic alteration using the Genome-Wide Human CytoScan HD Array (Affymetrix, CA, USA) according to the manufacture’s protocol. Scanned data was analyzed with Chromosome Analysis Suire v2.1 ((Affymetrix, CA, USA). SNP oligonucleotide microarray analysis was perfoemed using Affymetrix GnenChip Human Mapping 6.0 SNP array. Sample preparation, hybridization, and scanning were performed according to manufacture’s specifications (Affymetrix, CA, USA). Analyses were performed using the Genotyping Console v2.0 (Affymetrix) and Ingenuity Pathway Analysisv7.5 (Ingenuity Systems, CA, USA).

## Discussion

Generally, neoplastic meningitis is not uncommon with solid carcinoma, *e.g.*, breast cancer, lung cancer, and gastrointestinal cancer [6]; however, it is extremely rare to see the CSF dissemination of intraventricular malignant meningioma. To our knowledge, only eight such cases have been hitherto reported [7]. In these cases, tumor mass of meningioma in the brain or spinal cord was found transformed from low-grade meningioma to malignant meningioma, eventually resulting in the CSF dissemination of malignant tumor cells. In the present patient, the solitary tumor in the lateral ventricle contained mixed pathology of benign and malignant parts originated from the common cellular profile of the meningioma. Of note, unlike the previously reported cases, neuroimaging studies could not demonstrate any tumor mass suggestive of metastasis in the present patient. This may be one reason for the delay in the clinical diagnosis despite such widespread dissemination of tumor cells. CSF dissemination of malignant tumor cells may potentially cause a wide variety of neurological symptoms, such as hydrocephalus, cerebellar dysfunction, disturbances of multiple spinal nerve roots or the cauda equina, and cranial nerve palsy [4]. Conversely, brainstem dysfunction by CSF dissemination of malignant cells is rare; only a few reports are available in the literature in which Wallenberg syndrome or central hyperventilation was induced by CSF dissemination of malignant tumor cells [8, 9]. In the present patient, although marked and progressive decline of brainstem function was observed, an accurate diagnosis could not be made until the post-mortem examination, suggesting that it is by no means easy to make a prompt diagnosis of this type of clinical condition.

Clinically, peripheral facial nerve palsy, diplopia and unsteadiness of gait were the initial manifestations in the present patient. These symptoms, if they occur acutely, risk misleading the differential diagnoses towards more frequent clinical conditions, such as brainstem encephalitis or Fisher syndrome [10]. Unfortunately, CSF cytology, which was performed repeatedly, did not identify any malignant cells in the present patient. Although the specificity of CSF cytology is high, the sensitivity is low, between 45 and 95 % [11]. Reportedly, in cases of leptomeningeal metastasis, malignant cells were identified in the CSF in 54 % of cases on initial lumber puncture, and remained negative in 8 %, even after repeated examination [12]. The reason for the low specificity of the CSF cytology in leptomeningeal metastasis remains unclear regardless of extensive seeding of the malignant cells via the CSF. We speculate that the malignant cells may have a propensity to adhere easily to the meninges rather than floating freely in the CSF.

The pathology showed that the malignant part of this tumor was positive for cytokeratin. Cytokeratin, a known marker of metastatic carcinoma, was expressed only in cells of the malignant part. This finding did not rule out the diagnosis of meningioma because cytokeratin expression was also detected in 75 % of malignant meningiomas [13].

## Conclusion

Although it is certainly not easy to make a precise diagnosis of this type of clinical condition, it is clinically important to note that when seeing a patient with an intraventricular tumor in whom any neurological symptoms appeared progressively, the possibility should be kept in mind that CSF dissemination of tumor cells, after malignant transformation, might be the potential cause, even if CSF cytology is negative.

## Abbreviations

CSF, cerebrospinal fluid; CNS, central nervous system; MRI, magnetic resonance imaging; sIL2-R, Soluble interleukin 2-receptor; ACE, angiotensin-converting enzyme; ANCA, anti-neutrophil cytoplasmic antibody; AQP-4, anti-aquaporin-4; IVIg, intravenous immunoglobulins; DWI, diffusion-weighted image; FLAIR, fluid attenuated recovery; HE, hematoxylin-eosin; EMA, epithelial membrane antigen; GFAP, glial fibrillary acidic protein
